# Empathy and burnout in medical staff: mediating role of job satisfaction and job commitment

**DOI:** 10.1186/s12889-022-13405-4

**Published:** 2022-05-23

**Authors:** Zongpu Yue, Yang Qin, Ying Li, Jian Wang, Stephen Nicholas, Elizabeth Maitland, Cai Liu

**Affiliations:** 1 School of Management, Tianjin University of Traditional Chinese Medicine, Tianjin, 301617 China; 2 Dong Fureng Institute of Economic and Social Development, Wuhan University, No.54 Dongsi Lishi Hutong, Dongcheng District, Beijing, 100010 China; 3 Center for Health Economics and Management at School of Economics and Management, Wuhan University, Hubei Province, 299 Bayi Road, Wuchang District, Wuhan, 430072 China; 4 Australian National Institute of Management and Commerce, 1 Central Avenue Australian Technology Park, Eveleigh Sydney, NSW 2015 Australia; 5 School of Economics and School of Management, Tianjin Normal University, West Bin Shui Avenue, Tianjin, 300074 China; 6 Research Institute for International Strategies, Guangdong University of Foreign Studies, Baiyun Avenue North, Guangzhou, 510420 China; 7 Newcastle Business School, University of Newcastle, University Drive, Newcastle, NSW 2308 Australia; 8 School of Management, University of Liverpool, Chatham Building, Chatham Street, Liverpool, L697ZH England

**Keywords:** Medical staff, Job burnout, Empathy, SEM, Job satisfaction, Job Commitment

## Abstract

**Background:**

Burnout is a growing problem among medical staff worldwide and empathy has been described as an essential competence to attenuate burnout. Previous studies found job satisfaction and job commitment were affected by the empathy and associated with burnout. This study explores the effect and mechanism of empathy on burnout on medical staff and investigates the mediating role of job satisfaction and job commitment in the relationship between empathy and burnout among medical staff.

**Methods:**

Based on a self-administered questionnaire which included the Maslach Burnout Inventory (MBI) to measure burnout, 335 responses from medical staff in Tianjin City, China, yielded data on socio-demographic characteristics, empathy, burnout, job satisfaction and job commitment. Bivariate correlation and structured equation modeling (SEM) analyzed the relationships between empathy, job satisfaction, job commitment and burnout multi-group invariant analysis was used to evaluate whether the model was consistent across different type and level of hospitals and different job and employment type subgroups.

**Results:**

A total of 202 (60.3%) medical staff had low level burnout, 115 (34.3%) staff had the moderate level and 18 (5.4%) staff had the high level burnout. The results of the SEM showed that empathy not only had a direct negative effect on burnout ($\beta =-0.401, P<0.001)$, but also had an indirect impact through job satisfaction ($\beta =-0.373, P<0.001)$ and job commitment ($\beta =0.489, P<0.001)$. Job commitment was negatively associated burnout ($\beta =-0.513, P<0.001),$ but, unexpectedly, job satisfaction was positively associated with burnout ($\beta =0.177, P<0.001)$. The results also indicated the model was consistent across employment type ($\Delta {\chi }^{2}$ = 5.904, *p* > 0.05) and hospital type ($\Delta {\chi }^{2}$ = 7.748, *p* > 0.05), but was inconsistent across hospital level ($\Delta {\chi }^{2}$ = 42.930, *p* < 0.05) and job type ($\Delta {\chi }^{2}$ = 52.912, *p* < 0.05).

**Conclusions:**

Our results pointed out the important role that empathy plays in addressing burnout and revealed that managing job satisfaction and increasing the job commitment attenuated burnout. We recommend that the government should accelerate the reform of the resourcing of different hospital levels; facilitate hospital managers to implement additional training; and support hospitals to strengthen psychological testing and counseling to reduce medical staff burnout.

**Supplementary Information:**

The online version contains supplementary material available at 10.1186/s12889-022-13405-4.

## Background

Burnout, or when individuals experience chronic work-based stress for which the perceived job demands exceed personal and work-place resources, is common among medical workers [1]. Burnout was divided into three dimensions by Maslach and Jackson, comprising emotional exhaustion (feelings of being emotionally overextended and exhausted by one's work); depersonalization (an unfeeling and impersonal response toward colleagues and customers); and low personal-accomplishment (feelings of competence and successful achievement in one’s work) [2]. Research has identified burnout across a wide range of healthcare professions, including UK consultants [3], ear nose and throat surgeons [4], doctors [5] and psychiatric nurses [6]; Spanish oncology health professionals [7]; Singapore mental health workers [8]; and Australian clinical psychologists [9]. American doctors experience some of the fastest increasing burnout rates in the world, with one study estimating burnout at 40% in 2013, 46% in early 2015 and 65% in 2016 [10], and estimated burnout rates among U.S. palliative care staff varied widely between 9 and 61% [11]. Burnout among medical staff in Heilongjiang varied between 53 and 85% [12] and the Beijing young medical staff burnout rate was estimated at 65.1% [13]. One Chinese study found burnout reached 91.3% in the department of infectious disease in Wuhu [14] and 76.9% of Chinese physicians reported symptoms of burnout [15]. Burnout has been linked to a decline in work performance, high job separation and turnover rates, poorer health, staff sickness, mental illness, decreased patient satisfaction and increased medical errors [5, 16, 17].

The health care literature has explored the factors attenuating medical staff burnout, including empathy, job commitment and job satisfaction. Empathy is a unique psychological resource because of its impact both on patients and medical staff. Through the formation of personal relationships and enhanced communication with patients [18], empathy improves the doctor-patient relationship, resulting in better patient confidence in and compliance with treatment [19]. Since empathy helps to prevent physical and emotional workload exhaustion [18], doctors with high levels of empathy may be more resistant to burnout [20]. Many studies have investigated empathy as a predictive variable of burnout [19, 21] and burnout has been associated with a decline of empathy [22, 23]. For example, a study of primary care professionals in Spain found that higher levels of empathy were associated with lower burnout [21]. Studies have explored the relationship between empathy and three dimensions of burnout: empathy reduces job burnout in general [24]; empathy is negatively correlated with emotional exhaustion and depersonalization; and empathy is positively correlated with personal accomplishment [25]. We propose:

- H1: Empathy has a negative association with burnout.

In the burnout literature, many factors have been associated with burnout in medical staff, including sex, working hours, the presence of work-home conflict, coping strategies, staff perceptions, job commitment and job satisfaction [26]. Job satisfaction and job commitment have a major influence on job-related behaviors, such as job resignation, absenteeism, job performance and burnout [27, 28]. For example, burnout scale items have been found to be negatively and directly linked to job satisfaction [29]. Burnout dimensions, such as emotional exhaustion and low personal accomplishment have been shown to be negatively associated with job satisfaction [30]. By understanding job satisfaction and its relationship with burnout, employee behaviour can be positively influenced, which contributes to improved hospital performance and employee well-being [31]. The burnout literature has also argued that job commitment can insulate those who form a strong bond with their organization from experiencing burnout, with job commitment negatively associated with burnout [32–34]. As well a attenuating burnout, job commitment leads to fewer major medical errors and increases optimal patient care [5], and job commitment can also be harnessed to improve work and hospital performance. Empathy can impact both job satisfaction and job commitment. For example, empathy makes practicing medicine more meaningful, enhancing the sense of usefulness among medical staff, ensuring better job satisfaction and attenuating burnout [23, 35, 36] Similarly, empathy and burnout could be mediated by job commitment [37]. One study found that with more years of experience and greater job commitment, there is a sequential increase in nurses’ empathy, so variations in job commitmentcan improve the relationship between empathy and burnout [28, 37]. Raiziene et al. [38] found empathy was positively associated with occupational commitment, and they both were negatively associated with burnout. Previous studies found that job satisfaction influences job commitment significantly, we also propose the job satisfaction could influence job commitment [39]. We propose:

- H2a: Empathy has a positive effect on job satisfaction.
- H2b: Job satisfaction negatively mediates the relationship between empathy and job burnout.
- H3a: Empathy has a positive effect on job commitment
- H3b: Job commitment negatively mediates the relationship between empathy and job burnout

**Fig. 1:**
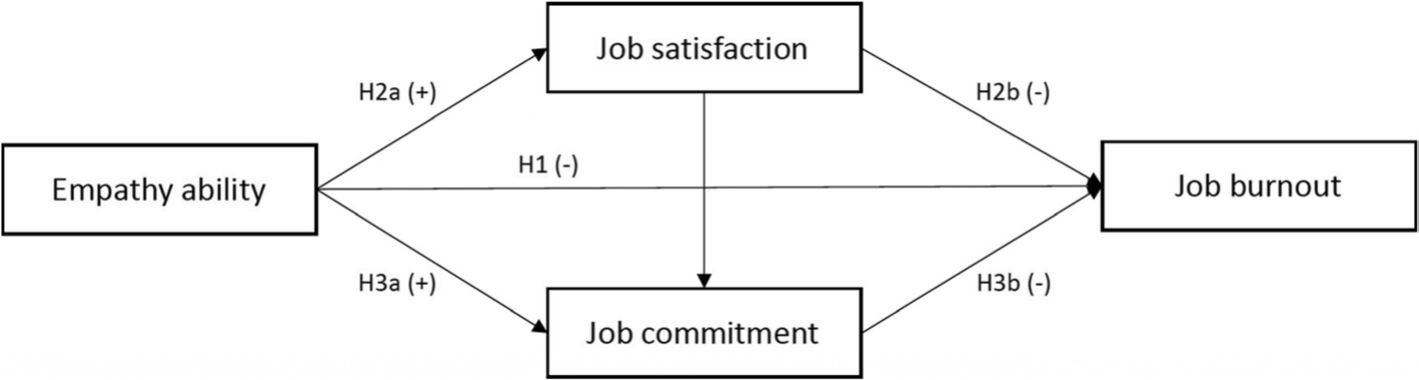
Mediated Empathy-Burnout Model

Based on the above discussion, Fig. 1 provides a diagrammatic representation linking burnout, empathy, job satisfaction and job commitment relationships, where empathy decreases burnout and job satisfaction and job commitment mediate the empathy-burnout relationship.

Previous research also found that the empathy ability and burnout level are different through several personal characteristics, such as work position, working experience and hospital types [20, 21, 40]. As a result, we also evaluate whether the model in Fig. 1 holds across hospital types, hospital levels, different positions and employment types and we propose:

- H4: The model in Fig. 1 is applicable to different hospital types, hospital levels, job positions and employment types.

Our study extends the empathy-burnout literature in several ways. Previous studies have mainly focused on the relationship between job burnout and empathy, or job commitment and job satisfaction and burnout. Few studies have focused on the mediating effect of variables on the empathy-burnout relationship. Constructing a mediated structural equation model, we extend the burnout literature by assessing whether job satisfaction and job commitment mediated the relationship between empathy and job burnout. Second, most burnout studies have collected data on one type of health professional, such as doctors or nurses, in a specific health setting, such as nursing homes or hospitals [41]. Our study collected data on all medical staff, including physicians, nurses and clinical pathologists, across different hospital levels and different hospital types in Tianjin, China. Finally, our study extends the literature about whether hospital type, hospital level, job positions and employment type affected the mediated model and if there were differences across hospital type and level and across job positions, managers should set different policies according to different hospital and medical groups to reduce burnout.

## Methods

### Participants and settings

Using cluster sampling, we conducted a pilot survey to assess the robustness and validity of our variables and the framing of our questions, before undertaking face-to-face and online surveys of medical professionals from six hospitals in Tianjin, China from July 1, 2019 to September 8, 2019. After the agreement of the human resource office in each hospital, we trained the staff on how to administer the questionnaire, the understanding of informed consent and the meaning of each questionnaire item. The questionaries were accompanied by an official letter from the human resource office in each hospital. Respondents could scan the QR code on their mobile phones to complete the survey or fill out paper questionnaires. In order to improve the accuracy of the survey responses, the survey purpose, method and the conceptual basis of each variable was explained to participants. The inclusion criteria of participants captured physicians, nurses, medical technicians, pharmacists, administrators and other medical personnel with more than 1 year of work experience engaged in medical and health work in Tianjin public medical and health institutions. All participants were volunteers, who gave informed consent to participate and were capable of completing the questionnaire independently. The responses by participants were anonymous.

The minimum sample size was calculated as 270 completed surveys [42–44]. Of the 350 distributed surveys, 15 uncompleted and inaccurate questionnaires were excluded, providing 335 valid responses, with a 95.71% effective response rate. Of the 335 valid questionnaires, 121 were completed online and 214 were completed using paper questionnaires. There were no significant differences in the responses between the online and paper questionnaires.

### Measures

The questionnaire composed five parts: job burnout, empathy, job satisfaction, job commitment and the respondent’s social-demographic characteristics, comprising age, sex, educational background, marital status, salary, workplace, professional titles and average daily working hours. The content of each scale is presented in Supplementary Table 1. Tianjin hospitals were categorized into three types: primary hospitals, equipped with less than 100 beds, only providing basic health services for a mainly local community; secondary hospitals, usually equipped with more than 100, but less than 500, beds, providing comprehensive medical services to several communities, with some teaching and scientific research activities; and tertiary hospitals, usually with more than 500 beds, considered a regional hospital providing high-level, specialty medical services to several districts, as well as performing higher level medical education and significant scientific research. Hospitals were also divided into two types, either specialist or general hospitals. Data on authorised employment type (entered the hospital or institution by passing a formal examination and registered in the China Organization Department and Ministry of Human Resources) and unauthorized employment type (all other employees) and job position were also collected.

#### Job burnout

To measure burnout, we employed the most commonly used burnout scale, the Maslach Burnout Inventory (MBI) [2, 45]. Comprising a total of 22 items, MBI contained three key dimensions, ‘Emotional Exhaustion’ (EE), ‘Depersonalization’ (DP) and ‘Low Personal Accomplishment’ (LPA), measured on a 7-point Likert scale (1-never to 7-every day). The EE and DP items were scored positively, with higher scores indicating a higher degree of job burnout, and the LPA items used the reverse scoring method, with the lower the score, the higher the degree of job burnout. In order to improve the effectiveness of the questionnaire, we did confirmatory factor analysis (CFA), which allowed us to delete the items whose coefficients were less than 0.6, yielding four EE variables and four DP variables and six LPA variables. According to previous research conducted in China, we defined high EE when the total score was more than 27; high DP when the score was more than 13; and high LPA when the score was less than 31. Next, we defined high level burnout when the levels were high in all three dimensions, medium level burnout when they were high in two levels, low level burnout when they were high in one level and no burnout when all levels were low [46].

#### Empathy

Empathy of medical staff was measured by the Chinese version of the Jefferson Scale Empathy – Health Professions (JSE-HP) [47]. The 20-item scale was divided into three dimensions: perspective-taking (PT), compassionate care (CC) and standing in patients’ shoes (SIPS). The items in PT were understanding the emotions of patients and their families, empathy, attention to body language; items in CC were the ability to understand and empathize with patients and help patients in timely manner, such as treating patients on the basis of understanding their emotions, paying attention to emotional changes when asking about their condition and considering patient's personal experience; and SIPS referred to the ability of medical staff to think from the patient's perspective, including treating the problem from the patient's perspective and thinking from the patient's perspective. As previous research [47] showed that JSE-HP measured empathy well, we did not modify these items. The scale included response categories from 1-completely disagree to 7-completely agree, with the CC and SIPS scale reverse scored. The total score was calculated, where a higher score indicated a high empathy ability.

#### Job satisfaction

Adopted from Zhang et al. [48, 49], the job description index (JDI) scale Chinese version measured the medical staff’s job satisfaction, based on five dimensions: the satisfaction with the job itself, promotion, salary, manager and partner. In the interviews with participants in the pilot survey, one common feedback was that the doctor-patient relationship, could influence them significantly, so we added “doctor-patient relationship” into the original scale. Therefore, the augmented JDI scale included a 6-dimensions scale, measured by a 5-point Likert scale (1-very dissatisfied to 5-very satisfied), with a higher score indicating the more job satisfied the respondent.

#### Job commitment

To measure job commitment, we used Meyer's three-dimensional scale, comprising ‘Emotional Commitment’(EM), ‘Continuous Commitment’ (CC) and ‘Normative Commitment’ (NC) items, and measured by a 5-point Likert scale (1—very dissatisfied to 5—very satisfied).

Cronbach's alpha values of each dimension in each scale and the values of the overall scale were all greater than 0.7, indicating good factor reliability. Most of the average variance extracted (AVE) values were greater than 0.5, indicating good aggregation validity of the model [50]. The details are presented in Supplementary Table 2 and Supplementary Table 3.

### Statistical analysis

#### Structure equation modelling

Structure equation modelling (SEM) refers to equations using parameters in the analysis of the observable, such as income or working hours, and latent variables, such as satisfaction and emotion. This study constructs a structural equation model of empathy and job burnout in medical staff and uses path analysis and diagrams to explain the internal relationships among variables to verify our hypotheses.

#### Mediating effect analysis

Mediating variables could explain the process of “how” and “why” between two variables. The influence of independent variables on dependent variables has both direct effects and indirect effects through mediating variables. Our research analyses and verifies the mediating effect of medical staff’s job satisfaction and job commitment between empathy and job burnout.

#### Multi-group analysis

This method was used to test whether the model we built is suitable for different population groups, which is whether the model is invariant among different sample groups. Additionally, it could verify whether a certain variable will have a moderating effect on the model. To test multi-group invariance, we divided our sample into three different hospital levels (primary hospital, secondary hospital and tertiary hospital); two hospital types (general hospital and specialty hospital); three job positions (physician, nurse and other medical staff); and two employment types (authorized employees and unauthorized employees).

### Statistical methods

Data entry and conversion was completed with EpiData 3.1, and SPSS 24.0 (IBM Corp, Armonk, NY, USA) and AMOS 23.0 (IBM Corp, Armonk, NY, USA) was used to analyze the data. Descriptive analyses explored the key social demographic factors and the degree of job burnout of medical staff. Next, a structural equation model (SEM) was specified to analyze empathy and job burnout, mediated by job satisfaction and job commitment. Based on the SEM, a multi-group equivalence analysis was conducted to determine the impact of different types and levels of hospital, job positions and employment types on the model. In the process of model fitting, 5000 Bootstrap tests to the mediating variables were conducted; observed variables with path coefficients less than 0.6 were deleted; and the modification index (MI) was used to optimize the model.

## Results

### Descriptive statistics

The socio-demographics of the 335 medical staff who completed the survey are presented in Table 1. Ranging from 18 to 66 years old, the average age was 37.65 $\pm$ 10.65 years; 45% were physicians (which requires a bachelor’s degree or above); the majority (54%) had achieved a bachelor’s degree and 5.1% had a PhD degree; 94% worked more than 6 h per day; and just over half (54.3%) earned more than RMB6000 per month, with 3.9% paid more than RMB15000 per month.

**Table 1:** Demographics characteristics of the sample (*N* = 335)

Variables	Group	Frequency	Percent
Sex	Male	178	53.1%
	Female	157	46.9%
Education background	Doctor and above	17	5.1%
	Master	65	19.4%
	Bachelor	181	54.0%
	Associate degree	73	21.5%
Marital status	Unmarried	81	24.3%
	Married	243	72.5%
	Divorced	7	2.1%
	Widowed	4	1.2%
Number of children	0	104	31.0%
	1	196	58.5%
	2 and above	35	10.4%
Hospital level	Tertiary	201	60.0%
	Secondary	76	22.7%
	Primary	58	17.3%
Hospital type Job position	General	233	69.6%
	Specialist	102	30.4%
	Physician	152	45.4%
	Nurse	75	22.7%
	Medical technician	25	7.5%
	Pharmacist	25	7.5%
	Administrator	34	10.1%
	Others	23	6.9%
Employment type	Authorized	252	75.2%
	Unauthorized	83	24.8%
Monthly income (RMB)	< 2000	18	5.4%
	2000 ~ 4000	45	13.4%
	4001 ~ 6000	90	26.9%
	6001 ~ 10,000	117	34.9%
	10,001 ~ 15,000	52	15.5%
	> 15,000	13	3.9%
Working hours per day	< 6	20	6.0%
	6—9	240	71.6%
	9—12	58	17.3%
	> 12	17	5.1%

There were 45 (13.4%) staff with low level burnout, 82 (24.48%) staff with moderate level burnout and 46 (13.73%) staff with high level burnout. Table 2 shows the breakdown in burnout percentages by job position. Medical staff were divided into physicians, nurse, medical technicians, pharmacists, administrators (including office manager and department director) and other medical staff (including clinical laboratory staff, interns and students). Low level burnout varied between 0.30% for pharmacists to 5.37% for physicians. Physicians experienced the highest rate of moderate level burnout (9.55%), followed by nurses (5.97%), with other medical staff (1.49%) displaying the lowest moderate burnout level. High level burnout ranged from 5.07% for other physicians, 2.99% for nurses to 1.79% for both administrators and other medical staff.

**Table 2:** Burnout level by the job position

	Physician	Nurse	Medical technician	Pharmacist	Administrator	Others
No Burnout (%)	25.37	8.96	2.39	4.78	4.18	2.69
Low Level (%)	5.37	4.78	0.60	0.30	1.49	0.90
Moderate Level (%)	9.55	5.97	2.99	1.79	2.69	1.49
High Level (%)	5.07	2.99	1.49	0.60	1.79	1.79

Table 3 shows the total score of job burnout of medical staff was 2.853 out of 7, which was lower than the median intensity (4 points) indicating that the overall extent of job burnout was low. All the burnout dimension sub-scores (emotional exhaustion 2.783, depersonalization 2.802, and low personal achievement 2.973) were also low. Pearson correlation analysis showed that all three dimensions of job burnout were positively correlated with overall job burnout (*p* < 0.01), and the highest correlation was emotional exhaustion (*r* = 0.933).

**Table 3:** Dimensions scores and total scores in job burnout

Dimensions	Number of items	Mean	SD	*Pearson* correlation Coefficient with job burnout	Burnout percent
Emotional exhaustion	4	2.783	1.498	.933**	22.09%
Depersonalization	4	2.802	1.484	.925**	23.58%
Low personal achievement	6	2.973	1.269	.834**	10.70%
Total job burnout	14	2.853	1.276	1.000	

Note:^**^p < 0.01

### Correlation analysis

Table 4 displays the correlation analysis results for medical staff empathy, job commitment, job satisfaction and job burnout. Empathy was significantly, and negatively, correlated with job burnout and job satisfaction (*p* < 0.01, *r* < 0) and positively correlated with job commitment (*p* < 0.01, *r* > 0). Job burnout was also significantly negatively correlated to job commitment and job satisfaction (*p* < 0.01, *r* < 0) and job commitment and job satisfaction were negatively correlated. (*p* < 0.01, *r* < 0).

**Table 4:** Correlation analysis among empathy ability, job burnout, job commitment and job satisfaction

Variables	Empathy	Job burnout	Job commitment	Job satisfaction
Empathy	1.000			
Job burnout	-.701**	1.000		
Job commitment	.637**	-.769**	1.000	
Job satisfaction	-.330**	.610**	-.512**	1.000

Note:*p* < 0.01

### Structural equation model

**Fig. 2:**
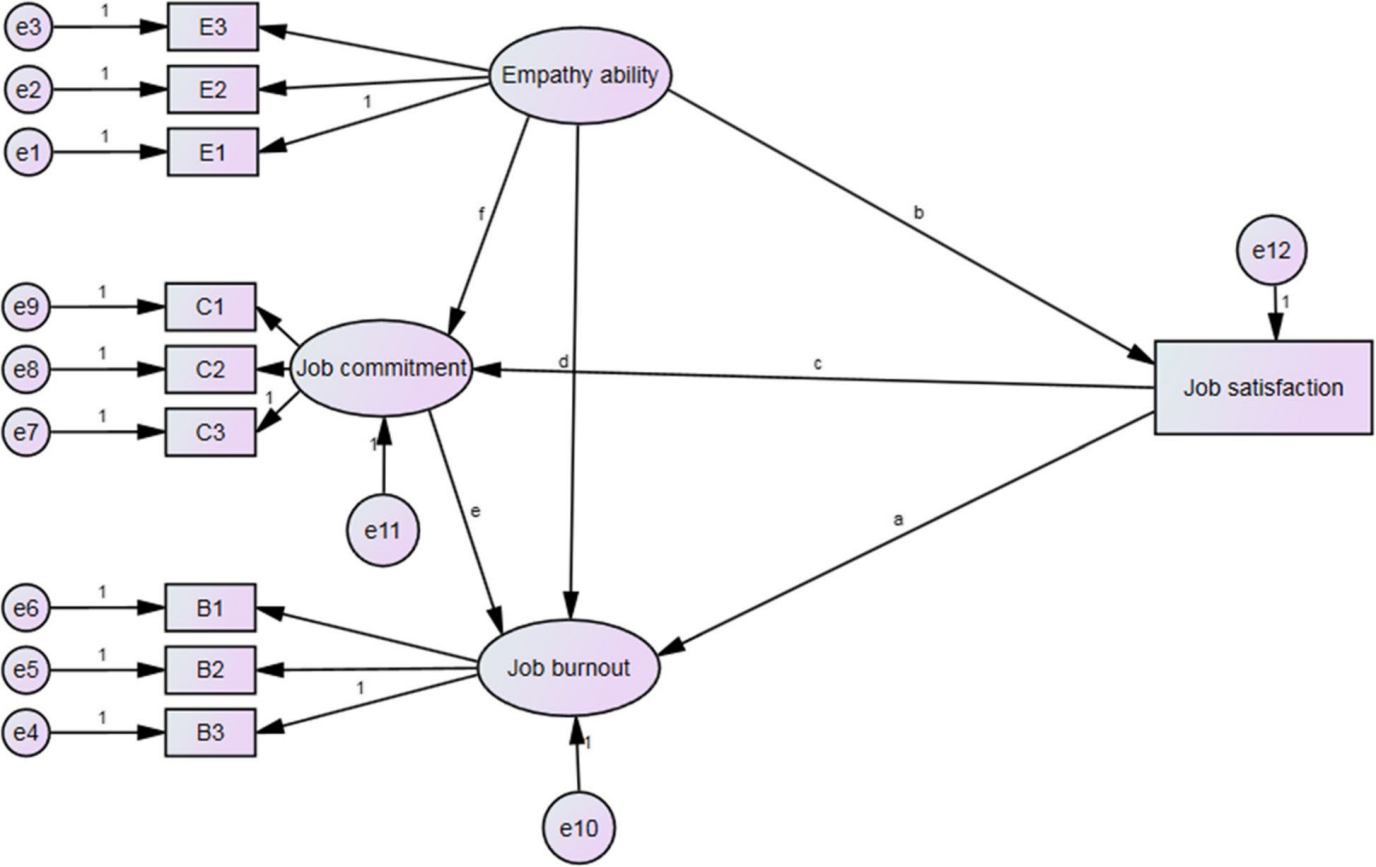
Structural equation hypothetical model of the relationship between empathy, job burnout, job satisfaction and job commitment

According to Fig. 1, empathy not only directly affects medical staff’s job burnout (H1), job satisfaction (H2a) and job commitment (H3a), but also has an indirect effect on job burnout through job satisfaction (H2b) and job commitment (H3b), where job satisfaction and job commitment play a mediating role in the relationship between empathy and job burnout. The six paths are shown in the full structural equation model Fig. 2: (1) Path a represents the total effect of empathy (E) to job burnout as specified in Hypothesis 1; (2) Path b represents the indirect effect of empathy to job satisfaction (H2a); Path c represents the indirect effect of empathy to job commitment (H3a); (3) Path d (H2b) and Path e (H3b) are the paths from potential mediating variable to dependent variable; (4) Path f specifies the relationship between the two mediating variables, representing the effect of job satisfaction on job commitment; and (5) E1-3, B1-3 and C1-3 are the dimensions of empathy, job commitment and job burnout, and (6) e1-9 represent the error terms of every dimension.

**Fig. 3:**
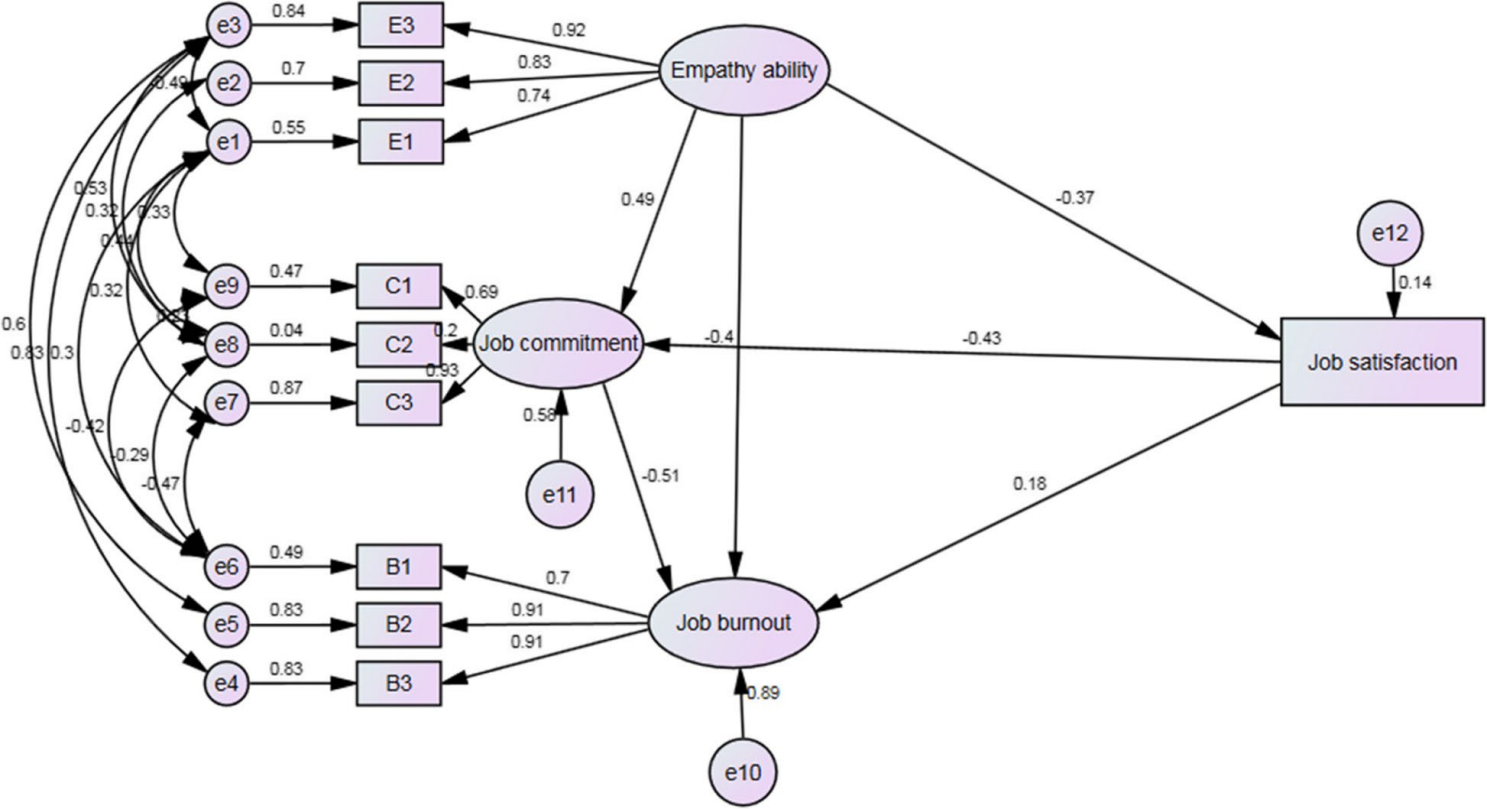
Revised structural equation model of the relationship between empathy, job burnout, job satisfaction and job commitment

We revised the model when some of the fitting indices did not meet the fitting criteria, indicating that the path map Fig. 2 was not ideal. According to the amendment advice given by AMOS, we added bidirectional arrows to improve model fit. The final fitting indices were ${\chi }^{2}$/*df* = 2.80, *AGFI* = 0.914, *GFI* = 0.937, *CFI* = 0.986, *TLI* = 0.964, *RMSEA* = 0.073, all of which meet the AMOS reference criteria [51]. The coefficients of the 6 paths are shown in Table 5 and the revised standard path map is displayed in Fig. 3, which shows the relationship and loading coefficient among empathy, job satisfaction, job commitment and job burnout.

**Table 5:** The test of path coefficient^a^ by MLE

Path	Non-standardized coefficient				Standardized coefficient
	*Unstd*	*S.E*	*C.R*	*P*	*Std*
Job satisfaction ← Empathy	-.355	.053	-6.717	**0.001**	-.373
Job commitment ← Empathy	.532	.056	9.428	**0.001**	.489
Job commitment ← Job satisfaction	-.487	.049	-9.976	**0.001**	-.426
Job burnout ← Empathy ability	-.619	.088	-7.043	**0.001**	-.401
Job burnout ← Job commitment	-.727	.098	-7.408	**0.001**	-.513
Job burnout ← Job satisfaction	.287	.067	4.288	**0.001**	.177

Note:^a^standardized path coefficient

As shown in Fig. 3, the standardized path coefficient of empathy to job burnout is -0.401 indicates that empathy is negatively correlated with job burnout, which confirms H1. The standardized path coefficient of empathy to job commitment is 0.489, revealing that job commitment was positively correlated with empathy, confirming H3a. But empathy had a negative effect on job satisfaction, where the coefficient was -0.373, which is the opposite of H2a. Job burnout decreased by 0.513 units for each additional unit of job commitment and increased by 0.177 units for each additional unit of job satisfaction. Additionally, the job commitment decreased by 0.426 units for each additional unit of job satisfaction.

Each path of the mediating variables was guided by 5000 repetitions of the Bias-corrected Bootstrap test and Percentile Bootstrap test. Supplementary Table 4 shows the significance test results of the pathways between empathy, job burnout, job satisfaction and job commitment. Regarding the pathway between empathy and job burnout, the mediation effect of job satisfaction on the relationship of empathy and job burnout was negative (*p*<0.01), 95% CI: (-0.159) - (-0.041), which was opposite to H2b. Job commitment had a negative mediation effect (*p*<0.01), 95% CI: (-0.544) - (0.269), which supports H3b.

### Multi-group invariance analysis

The multi-group invariance analysis tests whether the model is consistent across different subgroups, comprising different types of hospital, different levels of hospital, different medical jobs and different employment types. When there are (in)significant differences between different subgroup sample coefficients, the research model parameters are (in)variant, indicating that the multi-group model is (in)consistent across different subsamples.

Based on primary, secondary and tertiary hospital level, the results show that hospital level had a significant impact on the empathy and job burnout ($\Delta {\chi }^{2}$ = 42.930, *p* < 0.05). With regard to job satisfaction—job commitment path, the tertiary hospitals and secondary hospitals differed significantly compared to primary hospitals (*p* < 0.001). As shown in Supplementary Table 5, the influence of tertiary and secondary hospitals was greater than that of primary hospitals, and secondary hospitals were more influential than tertiary hospitals. On job satisfaction—job burnout path, secondary hospitals and primary hospitals displayed significant differences (*p* < 0.05), with the influence of the secondary hospital significantly greater than that of primary hospitals.

Next, we tested whether medical job type impacted empathy, job commitment and job satisfaction by dividing the sample into three medical occupation types: physician, nurse and other medical staff. The multi-group nested model indicated that different job type had a significant impact on empathy and job burnout model ($\Delta {\chi }^{2}$ = 52.912, *p* < 0.05). Supplementary Table 6 shows that there was a significant difference on the path of empathy—job burnout path among physicians and nurses (*p* < 0.01) and between nurses and other medical staff (*p* < 0.05). The nurse group had a significant impact on the prediction of job burnout (*p* < 0.001), while the physician group impact was not significant. The model showed that the influence of nurses was greater than that of physicians and other medical staff. On the job commitment—job burnout path, there was a significant difference between physicians and nurses (*p* < 0.001), between nurses and other medical staff (*p* < 0.05), and between physicians and other medical staff (*p* < 0.001). The influence of nurses was greater than that of physicians and other medical staff on the empathy—job commitment (*p* < 0.05). Further, the job satisfaction of physicians (*p* < 0.05) and other medical staff (*p* < 0.001) had a greater impact on job commitment than nurses’ job satisfaction, and other medical staff had more influence than physicians on job commitment (*p* < 0.001).

We also explored whether the employment type of medical staff impacted the model. Medical staff were divided into authorized and unauthorized employment types. First, the structural equation model (${\chi }^{2}$/*df* = 1.708, *NFI* = 0.966, *GFI* = 0.958, *CFI* = 0.985, *TLI* = 0.972, *RMSEA* = 0.046) revealed consistency across different employment types, which showed the empathy and job burnout model were not affected by employment type. Finally, hospitals were divided into two types, specialty hospitals and general hospitals. The structure weight model test showed a good fit (${\chi }^{2}$/*df* = 2.178, *NFI* = 0.957, *GFI* = 0.947, *CFI* = 0.976, *TLI* = 0.953, *RMSEA* = 0.059.), which means that the empathy and job burnout model was not affected by hospital type. These results show that the model was consistent across employment types and hospital type, which partly confirms H4. The model was not consistent across hospital level and job type.

## Discussion

The no burnout and low-level burnout rate was 61.79%, the moderate level burnout rate was 24.48% and the high-level burnout rate was 13.73%. Our burnout rates were lower than French mental healthcare low level burnout (77.5%); higher than the French moderate level burnout (17.8%); and much higher than the French high-level burnout (4.6%) [35] and consistent with the rates for Spanish physicians and nurses, where the low-level burnout rate was 58.8%, the moderate level was 37.5% while higher than the high level was 3.7% [21]. For medical staff in Heilongjiang, the moderate and high-level burnout rate was up to 84.62% and the Pakistani postgraduate trainees and house officers’ high-level burnout rate was 29.8% [40, 52], which are significantly higher than the results in our research.

### Influencing factors of burnout

We found significant differences in burnout according to age, marital status and working hours. Medical staff aged 56-66 years had the highest levels of job burnout. This finding is consistent with the argument that burnout is a gradual process, with the decline of job freshness and the increase of job challenges and difficulties, making medical staff more prone to burnout [53]. But the job burnout of 26-35 years old medical staff was higher than those 36-45 years old. This might reflect a bimodal function, where younger age medical staff are especially prone to burnout at the beginning period of their career, which makes participation in further education and training in managing workplace stress as important as training to improve work skills [54]. Job burnout of married medical staff was lower than that of the unmarried, probably related to family support attenuating job burnout [55]. Predictably, job burnout was highest for those working more than 12 hours, with long working hours, heavy workloads and repetitive work correlated with burnout [56]. Burnout was greater for those working below 6 hours per day than those who working 6-9 hours, which might reflect those working less than 6 hours per day not interacting with patients and their daily work may be more monotonous, such as dispensing drugs, or lacking technical content, such as administrative work [57]. Further, the salary of staff who working below 6 hours, such as pharmacist and administrative workers, was always less than doctors and nurses, with dissatisfaction with lower salary related to more burnout [58].

### Empathy and burnout

The bivariate spearman correlation analysis of job burnout, empathy, job satisfaction and job commitment showed empathy was negatively correlated with job burnout (*p*<0.01), which is consistent with H1 and previous research [59, 60]. Medical staff with higher empathy have a closer relationship with their patients, approach their job with more energy and enthusiasm, and are more likely to put themselves in the position of patients, possibly explaining why they were prone to low emotional exhaustion and depersonalization [61]. Medical staff who respond better to patient demands and solved patient problems will feel greater personal accomplishment, which can attenuate burnout.

### Job satisfaction and job commitment

The structural equation modelling tested for direct and indirect path effects. Empathy was positively correlated with job commitment (*p* < 0.01), which is consistent with the previous research [62] and confirms H3a. When medical staff have high empathy, they can better understand the patient and seek more measures to cure their patients, deepening their professional knowledge and enhancing their job commitment. We also found that job burnout was significantly negatively correlated with job commitment (*p* < 0.01), where increasing the job commitment of medical staff can shield health workers from burnout [63]. This is in line with H3b and through job commitment, medical staff can moderate their emotions [62], which reduces burnout, even when facing a long-term heavy workload. Job commitment provides a deeper understanding of their own behaviour when dealing with colleagues and communicating with patients and patients’ families, which attenuates burnout [63].

The mediating role of job satisfaction in the relationship of empathy and job burnout was consistent with previous research, which confirms H2b, although the correlation coefficients of previous results were greater than ours [64, 65], which can be explained by the sample selecting and methods we used [66]. First, medical staff in China work under a high workload and pressure, and this situation may cause the high burnout level [13] even if they are satisfied with their work. Second, previous research only considered the role of job satisfaction, while we not only researched the role of job satisfaction but also job commitment. However, the relationship between empathy and job satisfaction was negative, which is opposite to H2a. Empathy is one requirement of medical staff in the process of completing their daily work [67], but by using empathy to confront illness and death every day, the medical staff may be emotionally touched and overwhelmed by helplessness and powerlessness, causing dissatisfied with their work and working environment. The more doctors try to understand their patients, and the more responsible they feel, the more likely they are to criticize themselves and to become dissatisfied with their work.

### Hospital level and job position

Regarding the results of multi-group invariance analysis, hospital level and job position played a moderating role in our model. We found that the job satisfaction—job commitment and job satisfaction—job burnout paths for tertiary hospitals and secondary hospitals were significantly different (*p* < 0.001), while primary hospitals were not (*p* < 0.001). The heavy workload in primary hospitals, with a shortage of staff and the lack of social security and training opportunities, led to a decline in management efficiency and low service capabilities [68], so medical staff in primary hospital were not satisfied with their work. This result conforms to a nationwide survey of primary care doctors in China’s rural regions, which found that 70% of village doctors were unsatisfied with their income, had no hope of promotion and lacked social security after retirement [69].

The predicted effect of empathy on job burnout and job commitment on nurses was greater than that on physicians and other medical staff, which is consistent with previous research [70]. Empathy is more important in nurses' work than that of other medical staff, and nurses are better at thinking from the perspective of patients because of their training and their constant contact with patients [71], which contributes to job burnout. Also, the income gap between nurses and physicians was likely to lead to burnout [59]. We also found that the effect of job commitment—job burnout of physicians and other medical staff was stronger than nurses. Non-China studies have shown that there was no significant difference in job burnout among physicians, nurses and other medical staff [72], but Chinese research found that different types of medical staff revealed different attitudes towards patients. Compared to nurses who were more closely connected with patients, physicians tend to act in a non-personal way around patients, which promoted doctor depersonalization and job burnout [60]. Experiencing a high workload, physicians faced numerous patients daily, acting as both as the main diagnosticians of illness and decision-maker for treatment, creating high job commitment pressure, which can lead to job burnout.

### Employment type and hospital type

Our results found that employment type and hospital types did not influence the empathy—job burnout relationship, which indicates cross-group consistency. There are few previous SEM studies on the relationship between employment type and hospital types and empathy—job burnout. One study of Chinese medical staff found that authorized staff were more likely to suffer burnout from more work and higher requirements than the unauthorized staff [73]. However, we found that there was no significant difference between employment types. Due to the high employment pressure, even unauthorized employees made the same efforts as authorized staff to gain promotion, and their enthusiasm was not less than authorized staff. The fact that all medical staff both in general and specialty hospitals, faced a similar heavy workload, may explain the absence of significant differences between different types of hospitals, which confirmed H4 for these employees.

### Recommendations

Our results suggest that the government should increase the training of medical staff, paying special attention to the training of staff in primary health care facilities [14, 35]. As there was a shortage of medical staff, especially staff working in primary hospitals, government should increase staff levels. Second, further reforms should establish an integrated health care system to help relieve the workload on medical staff. Since 2009, China has undertaken a series of hospital reforms, including resourcing primary community health centres as gatekeepers, eliminating the drug mark-up policy, improving the health insurance system and promoting the efficiency of hospital management [74, 75]. Further reforms require the reallocation of resources between different hospital levels, especially correcting the concentration of resources in tertiary urban hospitals at the expense of rural primary health care facilities. Workload issues also arise from the over-use of tertiary and secondary hospitals when patients choose higher level hospitals even for minor medical problems, rather than using primary health care facilities as gatekeepers. Finally, hospitals should identify and support medical staff subject to burnout by strengthening psychological testing and intervention programs [21, 35, 73]. Since our results showed burnout varied by age, these support facilities should be age specific.

Hospital managers should enhance the job commitment of their medical staff by creating a good working environment, carrying out progressive education and giving medical staff training and learning opportunities. For example, advanced-study and experience exchange meetings would strengthen staff professional and technical levels, enhancing an individual’s coping ability by giving medical staff self-confidence to solve work problems. Higher levels of professional and technical ability would also promote job commitment. Through the establishment of a scientific shift-work systems, hospital managers should set the maximum daily working hours to avoid excess work hours and reduce the workload. At the same time, hospital managers can promote activities between different departments to create the formation of good cooperative relations among medical staff to improve their empathy ability.

### Limitations

Three limitations of the study should be addressed. First, although the SEM was used to quantitatively verify the relationship between variables, the use of cross-section data limited drawing causality conclusions. We also note that burnout might influence empathy, which requires long-term time series data and formal modelling as future research steps. Second, we only surveyed medical staff in Tianjin, and generalizing our results for other cities and regions, especially rural areas, requires further study. Third, our findings are largely based on the self-assessment questionnaires, with the risk that the participants’ answers might have been affected by response and social desirability bias.

## Conclusion

This study found that different types of medical staff in Tianjin experienced low level burnout rates between and 36 and 76%, moderate level burnout rates ranging between 20 to 43.5% and high-level burnout rates ranging from 4.0 to 8.7%. Our results revealed that empathy was negatively associated with burnout and job commitment had the negative effect on burnout. Job satisfaction had the positive effect on burnout, which was contrary to our hypothesis. Job satisfaction and job commitment played a mediating role in the relationship between empathy and burnout, which were strongly inter-correlated. We found the significant differences in burnout according to age, marital status and working hours. Additionally, the results indicated that the model was consistent across employment type and hospital type but was inconsistent across hospital level and job type. Based on our results, we recommend accelerating the reform of resourcing different hospital levels; facilitate hospital managers to implement additional training; and support hospitals to strengthen psychological testing and counselling.

## Supplementary Information

**Additional file 1:Supplementary Table 1.** Contents of three scales in the questionnaire. **Supplementary Table 2.** The reliability test results. **Supplementary Table 3.** The Person correlation results and AVE square root values. **Supplementary Table 4.** Mediating effect test analysis of paths by bootstrap. **Supplementary Table 5.** The path coefficient analysis of hospital levels. **Supplementary Table 6.** The path coefficient analysis of job position.

## Data Availability

All data are available from the corresponding author on reasonable request.

## Abbreviations

SD: Standard deviation
Std: Standardized path coefficient
Unstd: Unstandardized path coefficient
SE: Standard error
AGFI: Adjusted goodness of fit index
GFI: Goodness of fit
CFI: Comparative fit index
TLI: Tucker-Lewis index
RMSEA: Root mean square error of approximation
MLE: Maximum likelihood estimation
CR: Critical ration
CI: Confidence interval
