# Supplementary material for: Empathy and burnout in medical staff: mediating role of job satisfaction and job commitment
Source: BMC Public Health. 2022 May 23;22:1033. doi: 10.1186/s12889-022-13405-4 (PMC9125814; doi:10.1186/s12889-022-13405-4)
Supplement: Supplementary file 1 — Additional file 1:Supplementary Table 1. Contents of three scales in the questionnaire. Supplementary Table 2. The reliability test results. Supplementary Table 3. The Person correlation results and AVE square root values. Supplementary Table 4. Mediating effect test analysis of paths by bootstrap. Supplementary Table 5. The path coefficient analysis of hospital levels. Supplementary Table 6. The path coefficient analysis of job position. [file 12889_2022_13405_MOESM1_ESM.docx]

**Supplementary tables**

**Supplementary Table 1** Contents of three scales in the questionnaire

| **Scale** | **Content of each item** |
| --- | --- |
| Empathy ability (JSE-HP) | I try not to attention to my patients’ emotion in interviewing and history taking. |
|  | It is difficult for me to view things from patients’ perspective. |
|  | I believe that empathy is an important therapeutic factor in patients’ treatment. |
|  | I do not enjoy reading non-medical literature or the arts. |
|  | Patients feel better when I understand their feelings. |
|  | I think medical staff’s sense of humor contributes to a better clinical outcome. |
|  | Attentiveness to patients’ personal experiences does not influence treatment outcomes. |
|  | Understanding of the patients’ feelings and the feelings of patients’ families do not influence treatment outcomes. |
|  | Understanding body language is as important as verbal communication in my relationship with patient. |
|  | I consider asking patients about what is happening in their lives as an unimportant factor in understanding their physical complaints. |
|  | I do not allow myself to be touched by intense emotional relationships between my patients and their families. |
|  | Patients’ illnesses can be cured only by targeted treatment; therefore, emotional ties with patients do not have a significant influence in treatment outcomes. |
|  | I try to think like my patients in order to render better care. |
|  | I believe that emotion has no place in treatment of medical illness. |
|  | An important component of the relationship with my patients is my understanding of the emotional status of the patients and their families. |
|  | My understanding of my patients’ feeling gives me a sense of validation that is therapeutic in its own right. |
|  | I try to imagine myself in my patients’ shoes when providing care to them. |
|  | I try to understand what is going on in my patients’ mind by paying attention to their nonverbal cues and body language. |
|  | Because people are different, it is almost impossible for me to see things from my patients’ perspective. |
|  | Empathy is a therapeutic skill without which my success as a physician would be limited. |
| Job burnout (MBI) | I feel emotionally drained from my work. |
|  | I feel used up at the end of the workday. |
|  | I feel fatigued when I get up in the morning and have to face another day on the job. |
|  | I can easily understand how my patients or colleagues feel about things. |
|  | I feel I treat some patients as if they were impersonal objects. |
|  | Working with medical all day is really a strain for me. |
|  | I deal very effectively with the problems of medical work. |
|  | I feel burned out from my work. |
|  | I feel I’m positively influencing other people’s lives through my work. |
|  | I’ve became more callous towards people since I took this work. |
|  | I worry about this job is hardening me emotionally. |
|  | I feel very energetic. |
|  | I feel frustrated by my job. |
|  | I feel I’m working too hard on my job. |
|  | I don’t really care what happens to some patients. |
|  | Working with people directly puts too much stress on me. |
|  | I can easily create a relaxed atmosphere with my patients. |
|  | I feel exhilarated after working closely with my patients. |
|  | I have accomplished many worthwhile things in medical work. |
|  | I feel that I am at the end of my rope. |
|  | In my work I deal with emotional problems very calmly. |
|  | I feel patients blame me for some of their problems. |
| Job commitment | Medical working is important to my self-image. |
|  | I regret having entered the medical-working profession. |
|  | I am proud to be in the medical-working profession. |
|  | I dislike being a medical staff. |
|  | I do not identify with the medical-working profession. |
|  | I am enthusiastic about medical working. |
|  | I have put too much into the medical work to consider changing now. |
|  | Changing professions now would be difficult for me to do. |
|  | Too much of my life would be disrupted if I were to change my profession. |
|  | It would be costly for me to change my profession now. |
|  | There are no pressures to keep me from changing professions. |
|  | Changing professions now would require considerable personal sacrifice. |
|  | I believe people who have been trained in a profession have a responsibility to stay in that professions for a reasonable period of time. |
|  | I do not feel any obligation to remain in the nursing professions. |
|  | I feel a responsibility to the medical-working profession to continue in it. |
|  | Even if it were to my advantage, I do not feel that it would be right to leave medical work now. |
|  | I would feel guilty if I left medical work. |
|  | I am in medical working because of a sense of a loyalty to it. |
| Job satisfaction(JDI) | Satisfaction with job. |
|  | Satisfaction with present pay. |
|  | Satisfaction with promotion. |
|  | Satisfaction with supervision. |
|  | Satisfaction with co-worker. |
|  | Satisfaction with doctor-patient relationship. |

**Supplementary Table 2** The reliability test results

| Scale | Subscale | Cronbach’s Aplha in subscale | Cronbach’s Aplha in scale |
| --- | --- | --- | --- |
| MBI | EE | .869 | .927 |
|  | DP | .863 |  |
|  | LPA | .842 |  |
| JSPE-HP | PT | .852 | .938 |
|  | CC | .909 |  |
|  | SIPS | .739 |  |
| JDI | — | .862 | .862 |
| Mayer’s 3-Dimensional Scale | EM | .819 | .827 |
|  | CC | .541 | .818 |
|  | NC | .524 | .762 |

**Supplementary Table 3** The Person correlation results and AVE square root values

|  | AVE | Empathy ability | Job burnout | Job commitment | Job satisfaction |
| --- | --- | --- | --- | --- | --- |
| Empathy ability | .702 | **.838** |  |  |  |
| Job burnout | .747 | -.701 | **.864** |  |  |
| Job commitment | .501 | .637 | -.769 | **.708** |  |
| Job satisfaction | .520 | -.330 | .610 | -.512 | **.721** |

**Note: the value in bold is the AVE square root value**

**Supplementary Table 4** Mediating effect test analysis of paths by bootstrap

| Path | Point estimate | product of  coefficient | | Bias-corrected | | percentile | |
| --- | --- | --- | --- | --- | --- | --- | --- |
|  |  | *SE* | *Z* | *Lower* | *Upper* | *Lower* | *Upper* |
| JB ← Job satisfaction ← EA | -.102 | .030 | -3.400 | -.166 | -.048 | -.159 | -.041 |
| JB ← Job commitment ← EA | -.387 | .069 | -5.609 | -.562 | -.276 | -.544 | -.269 |
| JB ← Job commitment ← Job satisfaction ← EA | -.126 | .032 | -3.938 | -.201 | -.074 | -.195 | -.072 |
| Total mediating effect | -.615 | .077 | -7.987 | -.804 | -.486 | -.781 | -.477 |
| Total effect | -1.233 | .098 | -12.582 | -1.473 | -1.080 | -1.450 | -1.061 |

**Supplementary Table 5** The path coefficient analysis of hospital levels

| Path | Tertiary hospitals | | Secondary hospitals | | Primary hospitals | |
| --- | --- | --- | --- | --- | --- | --- |
|  | *Unstd.* | *Std.* | *Unstd.* | *Std.* | *Unstd.* | *Std.* |
| Job satisfaction ← EA | -.285^***^ | -.343 | -.344^*^ | -.280 | -.625^***^ | -.541 |
| Job commitment ← EA | .590^***^ | .565 | .362^**^ | .286 | .361^*^ | .456 |
| Job commitment ← Job satisfaction | -.502^***^ | -.400 | -.530^***^ | -.516 | -.129 | -.189 |
| JB ← EA | -.494^***^ | -.352 | -.727^**^ | -.389 | -1.086^***^ | -.638 |
| JB← Job commitment | -.731^***^ | -.543 | -.713^***^ | -.483 | -.803^**^ | -.373 |
| JB ← Job satisfaction | .319^***^ | .189 | .465^***^ | .306 | -.005 | -.003 |

**Note: *p < 0.05; **p < 0.01; ***p < 0.001**

**Supplementary Table 6** The path coefficient analysis of job position

| Path | Physician | | Nurse | | Other medical staff | |
| --- | --- | --- | --- | --- | --- | --- |
|  | *Unstd.* | *Std.* | *Unstd.* | *Std.* | *Unstd.* | *Std.* |
| Job satisfaction ← EA | -.3.40^***^ | -.360 | -.464^***^ | -.417 | -.331^***^ | -.387 |
| Job commitment ← EA | .547^***^ | .548 | .908^***^ | .655 | .348^***^ | .351 |
| Job commitment ← Job satisfaction | -.434^***^ | -.411 | -.181 | -.145 | -.747^***^ | -.644 |
| JB←EA | -.251 | -.164 | -1.185^***^ | -.697 | -.572^***^ | -.383 |
| JB ← Job commitment | -1.087^***^ | -.707 | -.215 | -.175 | -.962^***^ | -.640 |
| JB ← Job satisfaction | .268^*^ | .165 | .303^**^ | .199 | .120 | .069 |

**Note: *p < 0.05; **p < 0.01; ***p < 0.001**
